# Exploring the use of lithium for suicidality in acute settings: rationale, risks, and uncertainties

**DOI:** 10.47626/2237-6089-2024-0811

**Published:** 2025-03-27

**Authors:** Rafael Ramos Amaral, Pedro V. S. Magalhães

**Affiliations:** 1 Universidade Federal do Rio Grande do Sul Hospital de Clínicas de Porto Alegre Porto Alegre RS Brazil Hospital de Clínicas de Porto Alegre (HCPA), Universidade Federal do Rio Grande do Sul (UFRGS), Porto Alegre, RS, Brazil.; 2 UFRGS Faculdade de Medicina, Centro de Pesquisa Clínica Porto Alegre RS Brazil Programa de Pós-Graduação em Psiquiatria e Ciências do Comportamento, Faculdade de Medicina, Centro de Pesquisa Clínica, HCPA, UFRGS, Porto Alegre, RS, Brazil.

**Keywords:** Lithium, suicidality, mood disorders

## Abstract

Lithium's well-documented efficacy in preventing suicide over the long term prompts consideration of its potential for more rapid antisuicidal effects. However, evidence supporting such acute efficacy is limited. Weighing against its possible rapid benefits are significant concerns regarding lithium's side-effect profile – particularly toxicity, renal impairment, and weight gain – and the often necessary delay in achieving therapeutic levels. Moreover, the multifaceted nature of suicidality complicates identifying short-term outcomes and disentangling lithium's effects on suicidal thoughts from broader reductions in depressive symptoms. While lithium may remain invaluable for some high-risk patients in emergency settings, its routine use as a rapid-acting agent for acute suicidality is currently not well supported.

The quintessential mood stabilizer, lithium has had somewhat of a resurgence in the last few decades, partly due to evidence of effects that go beyond symptom improvement or even relapse prevention.^1^ Perhaps in an analogy to the current use of ketamine in emergency settings^2^ and owing to its capacity to prevent suicide in the long term,^3^ the use of lithium as a rapid-acting agent to relieve suicidality may be tempting in acute settings. Although at first glance this may seem like a reasonable strategy for depressed patients with suicidal thoughts, there are several reasons for examining this notion closely.

Firstly, as lifesaving as lithium can be when correctly indicated, it still carries risks that cannot be discounted, including dangerous toxicity, kidney damage, and weight gain.^4^ Secondly, running counter to the rapid-action goal, lithium is thought to have a delayed onset of antidepressant effects, possibly taking up to 6 to 8 weeks to be significant.^5^ The delay could be due to factors such as the need for gradual titration of dosages or a time lag required for the medication to achieve therapeutic serum concentrations. This is also consistent with lithium's mechanisms of action, which involve neurotransmitter signaling and cellular plasticity pathways through the modulation of enzymes like glycogen synthase kinase-3β (GSK3β).^6^ This uncertainty of the timespan for lithium antisuicidal actions has been raised before,^7^ and a protocol for a randomized, placebo-controlled multicenter trial to investigate lithium acute effects on suicidal ideation and behavior was published, but, to our knowledge, results have not been published and are not publicly available.^8^

Investigating the time course for drug-related actions on suicidality is challenging for several reasons. As a multifactorial and heterogeneous phenomenon, suicidality may be influenced by genetic, social, and circumstantial factors and present in different ways, ranging from eventual thoughts about dying to violent behavior towards oneself.^9^ As such, choice of outcome is critical in such studies and should account for both rapid and sustained changes in suicidality.^10^ Individual patient response can vary according to changes in drug absorption, distribution, metabolism and excretion, and there has been extensive investigation about differences between optimal and poor responses between patients.^6^ Improvement in underlying pathology can also be a determinant of lessening suicidality, and lithium effectiveness in reducing depressive symptoms in mood disorders can contribute to improvement in suicidality in the same way other interventions are suspected to do.^11,12^ A similar logic makes the use of lithium for suicidality in conditions where it is not thought to have substantial benefit, such as borderline personality disorder, even more questionable.

It has been suggested that evaluating an expanded spectrum of suicidality, such as behaviors and thoughts, can be a reasonable way to address the relative rarity of suicide as a short-term outcome.^13^ But suicidality in general and suicide only partly overlap, and lithium may lower suicides by other means, such as lethality, not necessarily directly affecting suicidal thoughts, for instance. Although sometimes proposed, broadly defined suicidality may not be a sufficiently valid proxy for suicide. Lithium is in a curiously inverse position: there is general agreement on its value in preventing suicide in mood disorders,^3,14^ but extrapolating these effects to alleviating suicidal thoughts regardless of diagnosis may not be warranted based on extant data. And testing lithium as a rapid-acting agent for suicidality may present its own set of methodological challenges as exposed above.

While we can say confidently that there is interest in drugs with acute antisuicidal effects and we perceive such use to be on the rise in local emergency and inpatient psychiatry services (sometimes regardless of diagnosis), we struggled to find empirical data on prescription pattern changes in these situations. Surely, drug prescription patterns are also variable and depend on context. We do not dispute the utility of having interventions for acute suicidality in medical and psychiatric emergency settings.^15^ There are also clinical situations where the use of lithium in the context of suicide risk in emergency settings is reasonable and based on firm evidence. Nevertheless, given the risk and uncertainty, we believe the rationale for offering lithium for acute suicidality in general in these settings is weak at this time and may ultimately provide an unwarranted sense of security regarding immediate suicide risk.
